# Existence of weak solutions of stochastic delay differential systems with Schrödinger–Brownian motions

**DOI:** 10.1186/s13660-018-1691-1

**Published:** 2018-04-26

**Authors:** Jianguo Sun, Liang Kou, Gang Guo, Guodong Zhao, Yong Wang

**Affiliations:** Department of Computer Science and Technology, Harbin Engineering University, Harbin, China

**Keywords:** Stochastic delay differential system, Schrödinger–Brownian motion, Schrödingerean Lyapunov functional

## Abstract

By using new Schrödinger type inequalities appearing in Jiang and Usó (J. Inequal. Appl. 2016:233, 2016), we study the existence of weak solutions of stochastic delay differential systems with Schrödinger–Brownian motions.

## Introduction

Controllability is one of the fundamental concepts in mathematical control theory, and it plays an important role in both deterministic and stochastic control systems. It is well known that controllability of stochastic delay differential systems is widely used in many fields of science and technology. The controllability of nonlinear deterministic systems represented by evolution equations in abstract spaces has been extensively studied by several authors (see [2, 3]). Stochastic delay differential system theory is a stochastic generalization of classical control theory.

In recent years, many mathematicians paid their attention to the existence of stochastic delay differential systems (see [1, 4–6]). However, to the best of our knowledge, little seems to be known about Schrödinger–Brownian motions for stochastic delay differential systems.

In this paper, we consider the stochastic delay differential system with Schrödinger–Brownian motion (SDDSs):

1.1 $$ \textstyle\begin{cases} \mathfrak{X}_{s}=x+\int_{0}^{s} b(s,\mathfrak{X}_{s},\mathfrak{Y}_{s}, \mathfrak{Z}_{s})\,ds+\int_{0}^{s} h(s,\mathfrak{X}_{s},\mathfrak{Y} _{s},\mathfrak{Z}_{s})\,d\langle B\rangle_{s} \\ \hphantom{\mathfrak{X}_{s}=}{}+\int_{0}^{s}\delta (s,\mathfrak{X}_{s},\mathfrak{Y}_{s}, \mathfrak{Z}_{s})\,d\mathfrak{B}_{s}; \\ \mathfrak{Y}_{s}=\Phi (\mathfrak{X}_{S})+\int^{S}_{s} b(s, \mathfrak{X}_{s},\mathfrak{Y}_{s},\mathfrak{Z}_{s})\,ds+\int^{S}_{s} h(s, \mathfrak{X}_{s},\mathfrak{Y}_{s},\mathfrak{Z}_{s})\,d\langle B\rangle _{s}-\int^{S}_{s} \mathfrak{Z}_{s}\,d\mathfrak{B}_{s} \\ \hphantom{\mathfrak{X}_{s}=}{}-(\mathfrak{K}_{S}-\mathfrak{K}_{s}), \end{cases} $$

where $\langle \mathfrak{B} \rangle $ is the quadratic variation of the Schrödinger–Brownian motion $\mathfrak{B}$. Under the Lipschitz assumptions on the coefficients *b*, *h*, and *σ*, Ren (see [7]) proved the well-posedness of such equations with the fixed-point iteration. Moreover, Yan (see [5]) studied the case when coefficients are integral–Lipschitz; Yan et al. (see [6]) considered the reflected GSDEs with some good boundaries; Jiang and Usó (see [1]) studied stochastic functional differential equation with infinite delay driven by Brownian motion.

It should be noticed that the coefficients of the equations are coupled with the solution of SSDDs (1.1). The question is whether there is a unique global solution $(\mathfrak{X}, \mathfrak{Y},\mathfrak{Z}, \mathfrak{K})$ for SSDDs (1.1). However, the coefficients that appeared in their equations are special. Precisely, *b*, *h*, *σ*, *f*, *g* do not contain *Z*. Moreover, unfortunately, they proved that SSDDs in their paper just have a unique local solution under Lipschitz condition. In this paper, we use the method presented in [1] and prove that there exists a unique weak solution for SSDDs (1.1) with some monotone coefficients.

The rest of this paper is organized as follows. In Sect. 2, we introduce some notions and results on the Schrödinger delay which are necessary for what follows. In Sect. 3, the existence theorem is provided.

## Preliminaries

In this section, we introduce some notations and preliminary results on the Schrödinger delay (see [8, 9] for more details).

Let $\Gamma_{S} = C_{0}([0,S ];R)$, the space of real-valued continuous functions on $[0,S]$ with $w_{0} = 0$, be endowed with the distance

2.1 $$ d\bigl(w^{1},w^{2}\bigr):=\sum_{N=1}^{\infty }2^{-N} \Bigl(\max_{0\le s\le N}\bigl\vert w_{s}^{1}-w_{s}^{2} \bigr\vert \Bigr)\land 1, $$

and let $\mathfrak{B}_{s}(w) = w_{s}$ be the canonical process. Denote by $\mathbb{F} := \{\mathcal{F}_{s}\}_{0\leq s\leq S} $ the natural filtration generated by $\mathfrak{B}_{s}$, $L^{0}(\Gamma_{S} )$ the space of all $\mathbb{F}$-measurable real functions. Let

$$ L_{ip}(\Gamma_{S}):= \bigl\{ \phi (\mathfrak{B}_{s_{1}} , \ldots,\mathfrak{B} _{s_{n}}):\forall n \ge 1, s_{1}, \ldots, s_{n} \in [0,S ], \forall \phi \in C_{b,L_{ip}} \bigl(R^{n}\bigr)\bigr\} , $$

where $C_{b,L_{ip}}(R^{n})$ denotes the set of bounded Lipschitz functions on $R^{n}$.

A sublinear functional on $L_{ip}(\Gamma_{S} )$ satisfies: for all $\mathfrak{X}$ and $\mathfrak{Y}\in L_{ip}(\Gamma_{S})$,

(I) Monotonicity:
  $$ \mathfrak{E}[\mathfrak{X}]\ge \mathfrak{E}[\mathfrak{Y}] $$
  if $\mathfrak{X}\le \mathfrak{Y}$.
(II) Constant preserving:
  $$ \mathfrak{E}[C]=C $$
  for $C\in R$.
(III) Sub-additivity:
  $$ \mathfrak{E}[\mathfrak{X}+\mathfrak{Y}]\le \mathfrak{E}[\mathfrak{X}]+ \mathfrak{E}[\mathfrak{Y}]. $$
(IV) Positive homogeneity:
  $$ \mathfrak{E}[\lambda \mathfrak{X}]=\lambda \mathfrak{E}[\mathfrak{X}] $$
  for $\lambda \geq 0$.

The triple $(\Gamma,L_{ip}(\Gamma_{S} ), \mathfrak{E})$ is called a sublinear expectation space and *E* is called a sublinear expectation.

### Definition 2.1

A random variable $\mathfrak{X}\in L_{ip}(\Gamma_{S})$ is Schrödinger normal distributed with parameters $(0,[\underline{ \sigma }^{2},\overline{\sigma }^{2}])$, i.e., $\mathfrak{X}\sim N(0,[\underline{\sigma}^{2},\overline{\sigma }^{2}])$ if, for each $\phi \in C_{b,L _{ip}}(R)$,

$$ u(s,x):=\mathfrak{E}\bigl[\phi (x+\sqrt{t}\mathfrak{X})\bigr] $$

is a viscosity solution to the following PDE:

$$ \textstyle\begin{cases} \frac{\partial u}{\partial s}+G\frac{\partial^{2} u}{\partial x^{2}}=0, \\ u_{s_{0}}=\phi (x) \end{cases} $$

on $R^{+}\times R$, where

$$ G(a):=\frac{1}{2}\bigl(a^{+}\overline{\sigma }^{2}-a^{-}\underline{\sigma }^{2}\bigr) $$

and $a\in R$.

### Definition 2.2

We call a sublinear expectation $\hat{\mathfrak{E}}:L_{ip}(\Gamma_{S}) \to R$ a Schrödinger expectation if the canonical process *B* is a Schrödinger–Brownian motion under $\hat{\mathfrak{E}}[\cdot]$, that is, for each $0 \le s \le t \le S$, the increment

$$ \mathfrak{B}_{s}-\mathfrak{B}_{s}\sim N\bigl(0,\bigl[\underline{\sigma }^{2}(s-s),\overline{ \sigma }^{2}\bigr](s-s)\bigr), $$

and for all $n > 0,0 \le s_{1}\le \cdots \le s_{n}\le S$, and $\varphi \in L_{ip}(\Gamma_{S})$,

$$ \hat{\mathfrak{E}}\bigl[\varphi (\mathfrak{B}_{s_{1}},\ldots, \mathfrak{B} _{s_{n-1}},\mathfrak{B}_{s_{n}}-\mathfrak{B}_{s_{n-1}}) \bigr]= \hat{\mathfrak{E}}\bigl[\psi (\mathfrak{B}_{s_{1}},\ldots, \mathfrak{B} _{s_{n-1}})\bigr], $$

where

$$ \psi (x_{1},\ldots,x_{n-1}):=\hat{\mathfrak{E}}\bigl[\varphi (x_{1}, \ldots,x_{n-1},\sqrt{s_{n}-s_{n-1}} \mathfrak{B}_{1})\bigr]. $$

We can also define the conditional Schrödinger expectation $\hat{\mathfrak{E}}_{s}$ of $\xi \in L_{ip}(\Gamma_{S} )$ knowing $L_{ip}(\Gamma {t})$ for $t \in [0,S]$. Without loss of generality, we can assume that *ξ* has the representation $\xi =\varphi ( \mathfrak{B}(s_{1}),\mathfrak{B}(s_{2})-\mathfrak{B}(s_{1}),\ldots, \mathfrak{B}(s_{n})-\mathfrak{B}(s_{n_{1}}))$ with $t = s_{i}$ for some $1 \le i \le n$, and we put

$$\begin{aligned} &\hat{\mathfrak{E}}_{s_{i}}\bigl[\varphi \bigl(\mathfrak{B}(s_{1}), \mathfrak{B}(s _{2})-\mathfrak{B}(s_{1}),\ldots, \mathfrak{B}(s_{n})-\mathfrak{B}(s _{n-1})\bigr)\bigr] \\ &\quad =\tilde{\varphi }\bigl(\mathfrak{B}(s_{1}), \mathfrak{B}(s_{2})- \mathfrak{B}(s_{1}),\ldots, \mathfrak{B}(s_{i})-\mathfrak{B}(s_{i-1})\bigr), \end{aligned}$$

where

$$ \tilde{\varphi }(x_{1},\ldots,x_{i})=\hat{\mathfrak{E}} \bigl[\varphi \bigl(x _{1},\ldots,x_{i}, \mathfrak{B}(s_{i+1})-\mathfrak{B}(s_{i}),\ldots, \mathfrak{B}(s_{n})-\mathfrak{B}(s_{n-1})\bigr)\bigr]. $$

For $p\ge 1$, we denote by $L_{G}^{p}(\Gamma_{S})$ the completion of $L_{ip}(\Gamma_{S})$ under the natural norm $\Vert X \Vert _{p,G}:=( \hat{\mathfrak{E}}[\vert X \vert ^{p}])^{\frac{1}{p}}$. $\hat{\mathfrak{E}}$ is a continuous mapping on $L_{ip}(\Gamma_{S} )$ endowed with the norm $\Vert \cdot \Vert _{1,G}$. Therefore, it can be extended continuous to $L_{G}^{1}(\Gamma_{S} )$ under the norm $\Vert X \Vert _{1,G}$.

Next, we introduce the Itô integral of Schrödinger–Brownian motion.

Let $M_{G}^{0} (0,S )$ be the collection of processes in the following form: for a given partition $\pi_{S} = \{s_{0}, s_{1}, \ldots, s_{N}\}$ of $[0,S ]$, set

$$ \eta_{s}(w)=\sum_{k=0}^{N-1} \xi_{k}(w)I_{[s_{k},s_{k+1})}(s), $$

where $\xi_{k}\in L_{ip}(\Gamma_{tk})$.

For $p\ge 1$, we denote by $H_{G}^{p}(0,S)$ and $M_{G}^{p}(0,S)$ the completion of $M_{G}^{0}(0,S)$ under the norms

$$ \Vert \eta \Vert _{H_{G}^{p}(0,S)}=\biggl\{ \hat{\mathfrak{E}}\biggl[\biggl( \int_{0} ^{S}\vert \eta_{s} \vert ^{2}\,ds\biggr)^{\frac{p}{2}}\biggr]\biggr\} ^{\frac{1}{p}}, $$

and

$$ \Vert \eta \Vert _{M_{G}^{p}(0,S)}=\biggl\{ \hat{\mathfrak{E}}\biggl[\biggl( \int_{0} ^{S}\vert \eta_{s} \vert ^{p}\,ds\biggr)\biggr]\biggr\} ^{\frac{1}{p}}, $$

respectively. It is easy to see that $H_{G}^{2}(0,S)=M_{G}^{2}(0,S)$. Following the method of Schrödingerean Lyapunov functional developed in [4], for each $\eta \in H_{G}^{p}(0,S)$ with $p\ge 1$, we can define the Itô integral $\int_{0}^{S}\eta_{s}\,d\mathfrak{B} _{s}$. Moreover, the following $B-D-G$ inequality holds.

### Lemma 2.3

*Let* $p\ge 2$ *and* $\eta \in M_{G}^{p}(0,S)$. *Then we have* (*see* [4])

$$ \underline{\sigma }^{p}c_{p}\hat{\mathfrak{E}}\biggl[ \biggl( \int -0^{S} \vert \eta_{s} \vert ^{2}\,ds \biggr)^{ \frac{p}{2}}\biggr]\le \hat{\mathfrak{E}}\biggl[\sup _{0\le s\le S}\biggl\vert \int_{0}^{s}\eta_{s}\,d\mathfrak{B}_{s} \biggr\vert ^{p}\biggr]\le \overline{\sigma }^{p}C_{p} \hat{\mathfrak{E}}\biggl[\biggl( \int_{o}^{S}\vert \eta_{s} \vert ^{2}\,ds \biggr)^{\frac{p}{2}}\biggr], $$

*where* $0 < c_{p} < C_{p} < \infty $ *are constants*.

Let

$$ S_{G}^{0}(0,S)=\bigl\{ h(s,\mathfrak{B}_{s_{1}\land t}, \ldots,\mathfrak{B} _{s_{n}\land t}):t,s_{1},\ldots,s_{n} \in [0,S],h\in C_{b,lib}\bigl(R^{n+1}\bigr) \bigr\} . $$

For $p\ge 1$ and $\eta \in S_{G}^{0}(0,S)$, set

$$ \Vert \eta \Vert _{S_{G}^{0}(0,S)}=\Bigl(\hat{\mathfrak{E}}\Bigl[ \sup _{0\le t \le S}\vert \eta_{s} \vert ^{p} \Bigr] \Bigr)^{\frac{1}{p}}. $$

Denote by $S_{G}^{p}(0,S) $ the completion of $S_{G}^{0}(0,S) $ under the norm $\Vert \cdot \Vert _{S_{G}^{p}(0,S)} $.

### Definition 2.4

Quadratic variation process of Schrödinger–Brownian motion defined by

$$ \langle \mathfrak{B} \rangle _{s}:=\mathfrak{B}_{s}^{2}-2 \int_{0}^{s}\mathfrak{B}_{s}\,d\mathfrak{B}_{s} $$

is a continuous and nondecreasing process.

For $\eta \in M_{G}^{0}(0,S)$, define

$$ \int_{0}^{S}\eta_{s}\,d\langle \mathfrak{B} \rangle _{s}=\sum_{j=0}^{N-1} \xi_{j}\bigl( \langle \mathfrak{B} \rangle _{s_{j+1}}- \langle \mathfrak{B} \rangle _{s_{j}}\bigr):M_{G}^{0}(0,S)\to L _{G}^{1}(\Gamma_{S}). $$

The mapping is continuous and can be extended to $M_{G}^{1}(0,S)$.

### Lemma 2.5

*Let* $p\ge 1$ *and* $\eta \in M_{G}^{p}(0,S)$. *Then we have* (*see* [8])

$$ \underline{\sigma }^{2} \hat{\mathfrak{E}}\biggl[\biggl( \int -0^{S} \vert \eta_{s} \vert \,ds\biggr)\biggr] \le \hat{\mathfrak{E}}\biggl[\sup_{0\le s\le S} \biggl\vert \int_{0}^{s}\eta_{s}\,d\langle B\rangle_{s}\biggr\vert \biggr] \le \overline{\sigma }^{p}C_{p}\hat{\mathfrak{E}}\biggl[\biggl(\int_{o}^{S} \vert \eta_{s} \vert \,ds \biggr) \biggr] $$

*and*

$$ \hat{\mathfrak{E}}\biggl[\sup_{0\le s\le S}\biggl\vert \int_{0}^{s}\eta_{s}\,d\langle B \rangle_{s} \biggr\vert ^{p}\biggr]\le \overline{\sigma }^{p}C_{p}^{\prime }\hat{\mathfrak{E}}\biggl[\biggl( \int_{o}^{S}\vert \eta_{s} \vert ^{p}\,ds \biggr) \biggr], $$

*where* $C^{\prime }_{p} >0$ *are constants independent of* *η*.

### Lemma 2.6

*Let* $\eta_{s}, \zeta_{s} \in M_{G}^{1} (0,S)$. *If* $\eta_{s}\le \zeta _{s} $ *for* $t \in [0,S] $, *then we have* (*see* [8])

$$ \int_{0}^{S}\eta_{s}\,d\langle B \rangle _{s}\le \int_{0}^{S} \zeta_{s}\,d\langle B \rangle _{s}. $$

## Main result and its proof

In this section, we consider the existence of the weak solution $(\mathfrak{X}, \mathfrak{Y}, \mathfrak{Z}, \mathfrak{K})$ for SSDDs (1.1). To state it, we need the following definitions.

### Definition 3.1

A quadruple process $(\mathfrak{X}, \mathfrak{Y},\mathfrak{Z}, \mathfrak{K})$ satisfying the above equations $(q.s.)$ is called a weak solution of equation (1.1) if $\mathfrak{X}, \mathfrak{Y},Z \in M_{G}^{2}(0, S)$, *K* is a decreasing process with $\mathfrak{K} _{0}=0$ and $\mathfrak{K}_{S} \in L_{G}^{2}(\Gamma_{S})$.

Let $u:=(x,y,z),A(s,u):=(-g(s,u),h(s,u),\sigma (s,u))$. $[\cdot, \cdot]$ denotes the usual inner product in a real number space and $\vert \cdot \vert $ denotes the Euclidean norm.

In the sequel, we will work under the following assumptions.

1. For $u\in R^{3}$, $\varepsilon >0$, $\Phi (x)\in L_{G}^{2}(\Gamma _{S})$, $f(\cdot,u)$, $g(\cdot,u)$, $b(\cdot,u)$, $h(\cdot,u)$, $\sigma (\cdot,u)\in M _{G}^{2}(0,S)$;
2. For $u^{1}$, $u^{2}\in R^{3}$, there exists a positive constant $C_{1}$ such that
  $$ \bigl\Vert f\bigl(s,u^{1}\bigr)-f\bigl(s,u^{2}\bigr) \bigr\Vert \lor \bigl\Vert b\bigl(s,u^{1}\bigr)-f\bigl(s,u^{2} \bigr) \bigr\Vert \lor \bigl\Vert A\bigl(s,u^{1}\bigr)-A\bigl(s,u^{2}\bigr) \bigr\Vert \le C_{1} \bigl\Vert u^{1}-u^{2} \bigr\Vert $$
  and
  $$ \bigl\Vert \Phi \bigl(x^{1}\bigr)-\Phi \bigl(x^{2}\bigr) \bigr\Vert \le C_{1}\bigl\Vert x^{1}-x^{2} \bigr\Vert ; $$
3. For $u^{1},u^{2}\in R^{3}$, there exists a positive constant *C* such that
  $$\begin{aligned}& \bigl[ A\bigl(s,u^{1}\bigr)-A\bigl(s,u^{2} \bigr),u^{1}-u^{2}\bigr] \le -C\bigl\Vert u^{1}-u^{2} \bigr\Vert ^{2}, \\& \bigl(-f\bigl(s,u^{1}\bigr)-(-f) \bigl(s,u^{2}\bigr)\bigr) \bigl(x^{1}-x^{2}\bigr) \le -C\bigl\Vert x^{1}-x^{2} \bigr\Vert ^{2}, \\& \bigl(b\bigl(s,u^{1}\bigr)-b\bigl(s,u^{2}\bigr) \bigr) \bigl(y^{1}-y^{2}\bigr)\le -C\bigl\Vert y^{1}-y^{2} \bigr\Vert ^{2} \end{aligned}$$
  and
  $$ \bigl(\Phi \bigl(x^{1}\bigr)-\Phi \bigl(x^{2}\bigr)\bigr) \bigl(x^{1}-x^{2}\bigr)\ge C\bigl(x^{1}-x^{2} \bigr)^{2}. $$

The following lemma plays a crucial role in establishing our main result.

### Lemma 3.2

*Suppose that* $\beta,\gamma,\lambda,\varphi \in M_{G}^{2}(0,S), \xi \in L_{G}^{2}(\Gamma_{S})$. *Then the following linear SSDDs*

3.1 $$ \textstyle\begin{cases}\,d\mathfrak{X}_{s}=(-\mathfrak{Y}_{s}+\beta_{s})\,ds+(-\mathfrak{Y}_{s}+ \gamma_{s})\,d\langle B \rangle _{s}+(-\mathfrak{Z}_{s}+\lambda _{s})\,d\mathfrak{B}_{s}, \\ d\mathfrak{Y}_{s}=(-\mathfrak{X}_{s}+\varphi_{s})\,ds+(-\mathfrak{X} _{s}+\phi_{s})\,d\langle B \rangle _{s}+\mathfrak{Z}_{s}\,d\mathfrak{B}_{s}+d\mathfrak{K}_{s}, \\ \mathfrak{X}_{0}=x,\mathfrak{Y}_{S}=\mathfrak{X}_{S}+\xi \end{cases} $$

*has a solution* $(\mathfrak{X},\mathfrak{Y},\mathfrak{Z},\mathfrak{K})$. *Moreover*, $\mathfrak{X}, \mathfrak{Y},Z \in M_{G}^{2}(0,S )$, *K* *is a decreasing Schrödinger martingale with* $\mathfrak{K}_{0} = 0$ *and* $\mathfrak{K}_{S} \in L_{G}^{2}(\Gamma_{S} )$.

### Proof

We consider the following linear SSDDs:

$$ \overline{Y}_{s}=\hat{\mathfrak{E}}_{s}\biggl[\xi - \int^{S}_{s}( \overline{Y}_{s}+ \varphi_{s}-\beta_{s})\,ds - \int^{S}_{s}(\overline{Y} _{s}+ \phi_{s}-\gamma_{s})\,d\langle B \rangle _{s}\biggr]. $$

By Lemma 2.3, it has an explicit solution

3.2 $$ \overline{Y}_{s}=\hat{\mathfrak{E}}_{s}\biggl[ \mathfrak{X}_{S}(\mathfrak{X} _{s})^{-1}\xi + \int^{S}_{s}(\beta_{s}- \varphi_{s} )\mathfrak{X}_{s}( \mathfrak{X}_{s})^{-1}\,ds+ \int^{S}_{s}(\gamma_{s}-\psi_{s} ) \mathfrak{X}_{s}(\mathfrak{X}_{s})^{-1}\,d\langle B \rangle _{s} \biggr], $$

where $\mathfrak{X}_{s}=\exp(-t- \langle B \rangle _{s})$.

Since $\{ \langle B \rangle _{s}\}_{t\in [0,S]}$ is an increasing process, then $\mathfrak{X}_{s}(\mathfrak{X}_{s})^{-1}<1 $ for $s\ge t$.

If we take the norm of $M_{G}^{2} (0, S )$ on both sides of (3.2), then we have

3.3 $$\begin{aligned} \int_{0}^{S}\hat{\mathfrak{E}}_{s}\vert \overline{Y}_{s} \vert ^{2} \leq & \int _{0}^{S}\hat{\mathfrak{E}}\biggl[\vert \xi \vert + \int^{S}_{s}\vert \beta_{s}- \varphi_{s} \vert \,ds+ \int^{S}_{s}\vert \gamma_{s}- \psi_{s} \vert \,d\langle B \rangle _{s}\biggr]^{2}\,ds \\ \leq &3 \int_{0}^{S}\biggl\{ \hat{\mathfrak{E}}\bigl[ \xi^{2}\bigr]+\hat{\mathfrak{E}} \sup_{t\in [0,S]}\biggl[ \int^{S}_{s}\vert \beta_{s}- \varphi_{s} \vert \,ds \biggr]^{2} \\ &+ \hat{\mathfrak{E}}\sup_{t\in [0,S]}\biggl[ \int^{S}_{s}\vert \gamma_{s}- \psi_{s} \vert \,d\langle B \rangle _{s} \biggr]^{2} \biggr\} \,ds \\ \leq &3S\biggl\{ \hat{\mathfrak{E}}\bigl[\xi^{2}\bigr]+S\hat{ \mathfrak{E}} \int^{S} _{s}\vert \beta_{s}- \varphi_{s} \vert ^{2}\,ds +\overline{\sigma }^{2} C_{2}^{ \prime }\hat{\mathfrak{E}} \int^{S}_{s}\vert \gamma_{s}- \psi_{s} \vert ^{2}\,ds \biggr\} \\ < &\infty, \end{aligned}$$

where in the last inequality, we have used Hölder’s inequality and Lemma 2.5. Thus we get $\overline{Y}_{s} \in M_{G}^{2}(0, S )$.

Since all Schrödinger martingales can be seen as conditional expectations, by the Schrödinger martingale representation theorem introduced in [10], there exists $\{\overline{Z}_{s}\}_{t\in [0,S]} \in M_{G}^{2}(0,S)$ and a decreasing Schrödinger martingale *K* with $\mathfrak{K}_{0} = 0$ and $\mathfrak{K}_{S} \in L_{G}^{2}(\Gamma_{S} )$ such that

3.4 $$\begin{aligned} \overline{Y}_{s}&=\xi - \int^{S}_{s}(\overline{Y}_{s}+ \varphi_{s}-\beta _{s})\,ds - \int^{S}_{s}(\overline{Y}_{s}+ \phi_{s}-\gamma_{s})\,d\langle B \rangle _{s} \\ &\quad {} - \int^{S}_{s}(2\overline{Z}_{s}- \lambda_{s})\,d\mathfrak{B}_{s}-( \mathfrak{K}_{S}- \mathfrak{K}_{s}). \end{aligned}$$

Thus the above equation has a solution $( \overline{Y}, \overline{Z}, \mathfrak{K})$. Moreover, *Y̅*, $\overline{Z}\in M_{G}^{2}(0,S)$ and *K* is a decreasing Schrödinger martingale with $\mathfrak{K}_{0} = 0$ and $\mathfrak{K}_{S}\in L_{G} ^{2}(\Gamma_{S})$. Then we consider the following SSDDs:

3.5 $$ \mathfrak{X}_{s}=x+ \int^{S}_{s}(-\mathfrak{X}_{s}- \overline{Y}_{s}+ \beta_{s})\,ds + \int^{S}_{s}(-\mathfrak{X}_{s}- \overline{Y}_{s}+\gamma _{s})\,d\langle B \rangle _{s} + \int^{S}_{s}(-\overline{Z}_{s}+ \lambda_{s})\,d\mathfrak{B}_{s}. $$

Since all of the coefficients satisfy the Lipschitz condition, then by Theorem 1.2 in [11] and Proposition 4.1 in [12], it has a unique weak solution $X\in S_{G}^{2}(0,S)$, which obviously belongs to a larger space $M_{G}^{2}(0,S)$.

Let $Y:= X+\bar{Y}$ and $Z=\bar{Z}$. Then we have

3.6 $$ \mathfrak{X}_{s}=x+ \int^{S}_{s}(-\mathfrak{Y}_{s}+ \beta_{s})\,ds + \int ^{S}_{s}(-\mathfrak{Y}_{s}+ \gamma_{s})\,d\langle B \rangle _{s} + \int^{S}_{s}(-\mathfrak{Z}_{s}+ \lambda_{s})\,d\mathfrak{B}_{s} $$

from (3.2), (3.3), (3.4), and (3.5).

Moreover, $Y\in M_{G}^{2}(0,S)$. We rewrite (3.4) as

3.7 $$ \overline{Y}_{s}=\bar{y}_{0}+ \int_{0}^{s}(\overline{Y}_{s}+\varphi _{s}-\beta_{s})\,ds + \int_{0}^{s}(\overline{Y}_{s}+ \phi_{s}-\gamma_{s})\,d\langle B \rangle _{s} + \int_{0}^{s}(2\overline{Z}_{s}- \lambda_{s})\,d\mathfrak{B}_{s}+\mathfrak{K}_{s}. $$

Combining (3.6) and (3.7), we have

3.8 $$ d\mathfrak{Y}_{s}=(-\mathfrak{X}_{s}+ \varphi_{s})\,ds+(-\mathfrak{X} _{s}+\phi_{s})\,d\langle B \rangle _{s}+\mathfrak{Z}_{s}\,d\mathfrak{B}_{s}+d \mathfrak{K}_{s}. $$

Thus $(\mathfrak{X}, \mathfrak{Y},\mathfrak{Z},\mathfrak{K})$ is a weak solution of (3.1) from (3.8). Moreover, $\mathfrak{X}$, $\mathfrak{Y}$, $\mathfrak{Z} \in M_{G}^{2}(0, S )$, and *K* is a decreasing Schrödinger martingale with $\mathfrak{K}_{0} = 0$ and $\mathfrak{K}_{S} \in L_{G}^{2}(\Gamma_{S} )$. □

The following assertion is the main result of the present paper.

### Theorem 3.3

*Let assumptions* (H1)–(H3) *hold*, *and for given* $\alpha_{0}\in [0,S), \forall \beta,\gamma,\lambda,\varphi \in M_{G}^{2}(0,S)$ *and* $\xi \in L_{G}^{2}(\Gamma _{S})$. *Then*

(I) *the following SSDDs* (1.1) *has a weak solution* $( \mathfrak{X}^{\alpha_{0}},\mathfrak{Y}^{\alpha_{0}},\mathfrak{Z}^{ \alpha_{0}},\mathfrak{K}^{\alpha_{0}})$, *where* $\mathfrak{X}^{\alpha _{0}},\mathfrak{Y}^{\alpha_{0}},\mathfrak{Z}^{\alpha_{0}} \in M_{G} ^{2}(0,S)$, $\mathfrak{K}^{\alpha_{0}}$ *is a decreasing Schrödinger martingale with* $\mathfrak{K}_{0}^{\alpha_{0}}$ *and* $\mathfrak{K}_{S} ^{\alpha_{0}}\in L_{G}^{2}(\Gamma )$.
(II) *There exists a constant* $\delta_{0} \in (0, 1)$ *such that*, *for all* $\alpha \in [\alpha_{0},\alpha_{0}+\delta_{0}]$, *equation* (3.7) *has an adapted solution* $(\mathfrak{X}^{\alpha },\mathfrak{Y}^{\alpha },\mathfrak{Z}^{\alpha },\mathfrak{K}^{\alpha })$.
(III) $(\mathfrak{X}^{\alpha }, \mathfrak{Y}^{\alpha }, \mathfrak{Z} ^{\alpha })\in M_{G}^{2}(\Gamma )$. *And* $\mathfrak{K}^{\alpha }$ *is a decreasing process with* $\mathfrak{K}_{0}^{\alpha }$ *and* $\mathfrak{K}_{S}^{\alpha }\in L_{G}^{2}(\Gamma )$.

### Proof

We define, for any given $\alpha \in [0,1]$,

$$\begin{aligned}& b^{\alpha }(s, x, y, z)=\alpha b(s, x, y, z)+(1-\alpha ) (-y), \\& \sigma^{\alpha }(s, x, y, z)=\alpha \sigma (s, x, y, z)+(1- \alpha ) (-z), \\& h^{\alpha }(s, x, y, z)=\alpha h(s, x, y, z)+(1-\alpha ) (-y), \\& (-f)^{\alpha }(s, x, y, z)=-\alpha f(s, x, y, z)+(1-\alpha ) (-x), \\& (-g)^{\alpha }(s, x, y_{)}z)=-\alpha g(s, x, y_{)}z)+(1-\alpha ) (-x), \\& \Phi^{\alpha }(x)=\alpha \Phi (x)+(1-\alpha )x. \end{aligned}$$

We set $u^{0}=(\mathfrak{X}^{0}, \mathfrak{Y}^{0}, \mathfrak{Z}^{0})=0$ and solve iteratively the following equations:

3.9 $$ \textstyle\begin{cases} \mathfrak{X}_{s}^{i+1}=x+\int_{0}^{t}[b^{\alpha_{0}}(s, u_{s+1})+ \delta (\mathrm{y}_{s}+b(s, u_{s}^{i}))+\beta_{s}]\,ds \\ \hphantom{\mathfrak{X}_{s}^{i+1}=}{}+\int_{0}^{t}[h^{\alpha_{0}}(s, u_{s}^{i+1})+\delta ( \mathfrak{Y}_{s}^{i}+h(s, u_{s}^{i}))+\gamma_{s}]\,d\{\mathfrak{B}\} _{s} \\ \hphantom{\mathfrak{X}_{s}^{i+1}=}{}+\int_{0}^{t}[\sigma^{\alpha_{0}} (s,u_{s}^{i+1})+\delta (\mathfrak{Z}_{s}^{i}+\sigma (s,u_{s}^{i}))+\gamma_{s}]\,d\mathfrak{B} _{s}; \\ \mathfrak{Y}_{s}^{i+1}=[\Phi^{\alpha_{0}}(\mathfrak{X}_{S}^{i+1})+ \delta (-\mathfrak{X}_{S}^{i}+\Phi (\mathfrak{X}_{S}^{i}))+\xi ] \\ \hphantom{\mathfrak{X}_{s}^{i+1}=}{}-\int_{s}^{S}[(-f)^{\alpha_{0}}(s, u_{s}^{i+1})+\delta (\mathfrak{X}_{s}^{i}-f(s, u_{s}^{i}))+\varphi_{s}]\,ds \\ \hphantom{\mathfrak{X}_{s}^{i+1}=}{}-\int_{s}^{S}[(-g)^{\alpha_{0}}(s, u_{s}^{i+1})+\delta (\mathfrak{X}_{s}^{i}-g(s, u_{s}^{i}))+\psi_{s}]\,d\langle \mathfrak{B} \rangle _{s} \\ \hphantom{\mathfrak{X}_{s}^{i+1}=}{} -\int_{s}^{S}\mathfrak{Z}_{s}^{i+1}\,d\mathfrak{B}_{s}-( \mathfrak{K}_{S}^{i+1}-\mathfrak{K}_{s}^{i+1}), \end{cases} $$

where $u^{i}=(\mathfrak{X}^{i}, \mathfrak{Y}^{i}, \mathfrak{Z}^{i}), i=0,1,2, \ldots $ .

Actually, by iterating and the assumptions of the theorem, it is easy to see that this equation has at least one solution $(\mathfrak{X} ^{i}, \mathfrak{Y}^{i}, \mathfrak{Z}^{i}, \mathfrak{K}), \mathfrak{X}^{i}, \mathfrak{Y}^{i}, \mathfrak{Z}^{i}\in M_{G}^{2}(0, S)$, $\mathfrak{K}^{i}$ is a decreasing Schrödinger martingale with $\mathfrak{K}_{0}^{i}=0$ and $\mathfrak{K}_{S}^{i}\in L_{G}^{2}( \Gamma )$ for each $i=0,1,2, \ldots $ .

We set

$$\begin{aligned}& \hat{u}^{i+1}=\bigl(\hat{\mathfrak{X}}^{i+1},\hat{\mathfrak{Y}}^{i+1}, \hat{\mathfrak{Z}}^{i+1} \bigr)=u^{i+1}-u^{i}, \\& \hat{f}\bigl(s, u_{s}^{i}\bigr)=f\bigl(s, u_{s}^{i}\bigr)-f\bigl(s, u_{s}^{i-1} \bigr), \qquad \hat{b}\bigl(s, u_{s}^{i}\bigr)=b\bigl(s, u_{s}^{i}\bigr)-b\bigl(s, u_{s}^{i-1} \bigr), \\& \hat{\mathfrak{K}}_{s}^{i+1}=\mathfrak{K}_{s}^{i+1}- \mathfrak{K}_{s} ^{i}\quad \mbox{and}\quad A^{\alpha }= \bigl((-g)^{\alpha }, h^{\alpha }, \sigma^{\alpha }\bigr). \end{aligned}$$

Using the convexity of the norm and (3.4), we deduce that

$$\begin{aligned} \Vert u_{n+1}-\hat{u} \Vert ^{2}& \le (1-\mathfrak{X}_{n})\Vert u _{n}-\hat{u} \Vert ^{2}+\mathfrak{X}_{n}\Vert v_{n}-\hat{u}\Vert ^{2} \\ &\le (1-\mathfrak{X}_{n})\Vert u_{n}-\hat{u} \Vert ^{2} \\ &\quad {}+ \mathfrak{X}_{n}\biggl\Vert {-}\mathfrak{Y}_{n} \hat{u}+(1-\mathfrak{Y} _{n})\biggl[u_{n}-\frac{\mathfrak{Z}_{n}}{1-\mathfrak{Y}_{n}}f^{*}fu_{n} -\biggl( \hat{u}- \frac{\mathfrak{Z}_{n}}{1-\mathfrak{Y}_{n}}f^{*}f\hat{u}\biggr)\biggr]\biggr\Vert ^{2} \\ & \le (1-\mathfrak{X}_{n})\Vert u_{n}-\hat{u} \Vert ^{2}+ \mathfrak{X}_{n}\mathfrak{Y}_{n}\Vert {-}\hat{u} \Vert ^{2}+(1- \mathfrak{Y}_{n}) \mathfrak{X}_{n}\biggl[\Vert u_{n}-\hat{u} \Vert ^{2} \\ & \quad {} + \frac{\mathfrak{Z}_{n}}{1-\mathfrak{Y}_{n}}\biggl(\frac{\mathfrak{Z}_{n}}{1- \mathfrak{Y}_{n}}-\frac{2}{\rho (f^{*}f)}\biggr)\bigl\Vert f^{*}fu_{n}-f^{*}f \hat{u}\bigr\Vert ^{2}\biggr] \\ & \le \Vert u_{n}-\hat{u} \Vert ^{2}+ \mathfrak{X}_{n}\mathfrak{Y} _{n}\Vert {-}\hat{u} \Vert ^{2} \\ & \quad {} + \mathfrak{X}_{n}\mathfrak{Z}_{n}\biggl(\frac{\mathfrak{Z}_{n}}{1- \mathfrak{Y}_{n}}- \frac{2}{\rho (f^{*}f)}\biggr)\bigl\Vert f^{*}fu_{n}-f^{*}f \hat{u}\bigr\Vert ^{2}, \end{aligned}$$

which implies that

$$\begin{aligned} \mathfrak{X}_{n}\mathfrak{Z}_{n} \biggl(\frac{2}{\rho (f^{*}f)}-\frac{ \mathfrak{Z}_{n}}{1-\mathfrak{Y}_{n}}\biggr)\bigl\Vert f^{*}fu_{n}-f^{*}f \hat{u}\bigr\Vert ^{2} &\le \Vert u_{n}-\hat{u} \Vert ^{2}-\Vert u _{n+1}-\hat{u} \Vert ^{2}+ \mathfrak{X}_{n}\mathfrak{Y}_{n}\Vert {-}\hat{u} \Vert ^{2} \\ &\le \Vert u_{n+1}-u_{n} \Vert \bigl(\Vert u_{n}-\hat{u} \Vert \\ &\quad {}+ \Vert u_{n+1}-\hat{u} \Vert \bigr)+\mathfrak{X}_{n} \mathfrak{Y}_{n} \Vert {-}\hat{u} \Vert ^{2}. \end{aligned}$$

Since

$$\begin{aligned}& \lim_{n\rightarrow \infty }\inf \mathfrak{X}_{n}\mathfrak{Z} _{n} \biggl(\frac{2}{\rho (f^{*}f)}-\frac{\mathfrak{Z}_{n}}{1-\mathfrak{Y} _{n}}\biggr)>0, \\& \lim_{n\rightarrow \infty }\mathfrak{Y}_{n}=0 \end{aligned}$$

and

$$ \lim_{n\rightarrow \infty }\Vert u_{n+1}-u_{n} \Vert =0, $$

we have

3.10 $$ \lim_{n\rightarrow \infty }\bigl\Vert f^{*}fu_{n}-f^{*}f \hat{u}\bigr\Vert =0. $$

Applying Lemma 2.6 and the property of the projection $P_{S_{i}}$, one can easily show that

3.11 $$\begin{aligned} &\Vert v_{n}-\hat{u} \Vert ^{2} \\ &\quad =\bigl\Vert P_{S_{i}}\bigl[(1-\mathfrak{Y}_{n})u_{n}-\mathfrak{Z}_{n}f^{*}fu _{n}\bigr]-P_{S_{i}}[\hat{u}-tG*G\hat{u}\bigr\Vert ^{2} \\ &\quad \le \bigl\langle (1-\mathfrak{Y}_{n})u_{n}-\mathfrak{Z}_{n}f^{*}fu _{n}-\bigl(\hat{u}-\mathfrak{Z}_{n}f^{*}f\hat{u}\bigr),v_{n}-\hat{u}\bigr\rangle \\ &\quad =\frac{1}{2}\bigl(\bigl\Vert u_{n}-\mathfrak{Z}_{n}f^{*}fu_{n}-\bigl(\hat{u}-\mathfrak{Z}_{n}f^{*}f\hat{u}\bigr)-\mathfrak{Y}_{n}u_{n} \bigr\Vert ^{2}+ \Vert v_{n}-\hat{u}\Vert ^{2} \\ & \quad \quad {}-\bigl\Vert (1-\mathfrak{Y}_{n})u_{n}-\mathfrak{Z}_{n}f^{*}fu_{n}-\bigl( \hat{u}-\mathfrak{Z}_{n}f^{*}f\hat{u}\bigr)-v_{n}+\hat{u}\bigr\Vert ^{2}\bigr) \\ &\quad \le \frac{1}{2}\bigl(\Vert u_{n}-\hat{u}\Vert ^{2}+2\mathfrak{Y}_{n} \Vert {-}u_{n}\Vert \bigl\Vert u_{n}-\mathfrak{Z}_{n}f^{*}fu_{n}- \bigl( \hat{u}-\mathfrak{Z}_{n}f^{*}f\hat{u}\bigr) - \mathfrak{Y}_{n}u_{n}\bigr\Vert \\ & \quad \quad {}+\Vert v_{n}-\hat{u}\Vert ^{2}-\bigl\Vert u_{n}-v_{n}-\mathfrak{Z} _{n}f^{*}f(u_{n}- \hat{u})-\mathfrak{Y}_{n}u_{n}\bigr\Vert ^{2} \bigr) \\ &\quad \le \frac{1}{2}\bigl(\Vert u_{n}-\hat{u}\Vert ^{2}+\mathfrak{Y}_{n}M+ \Vert v_{n}-\hat{u} \Vert ^{2}-\Vert u_{n}-v_{n}\Vert ^{2} \\ &\quad \quad {} +2\mathfrak{Z}_{n} \bigl\langle u_{n}-v_{n},f^{*}f(u_{n}- \hat{u}) \bigr\rangle \\ & \quad \quad {}+2\mathfrak{Y}_{n} \langle u_{n},u_{n}-v_{n} \rangle -\bigl\Vert \mathfrak{Z} _{n}f^{*}f(u_{n}- \hat{u})+\mathfrak{Y}_{n}u_{n}\bigr\Vert ^{2} \bigr) \\ &\quad \le \frac{1}{2}\bigl(\Vert u_{n}-\hat{u}\Vert ^{2}+\mathfrak{Y}_{n}M+ \Vert v_{n}-\hat{u} \Vert ^{2}-\Vert u_{n}-v_{n}\Vert ^{2} \\ & \quad \quad {}+2\mathfrak{Z}_{n}\Vert u_{n}-v_{n} \Vert \bigl\Vert f^{*}f(u_{n}- \hat{u})\bigr\Vert +2 \mathfrak{Y}_{n}\Vert u_{n}\Vert \Vert u_{n}-v _{n}\Vert \bigr) \\ &\quad \le \Vert u_{n}-\hat{u}\Vert ^{2}+ \mathfrak{Y}_{n}M-\Vert u_{n}-v _{n}\Vert ^{2} \\ & \quad \quad {} -\Vert u_{n}-v_{n}\Vert ^{2}+4 \mathfrak{Z}_{n}\Vert u_{n}-v_{n}\Vert \bigl\Vert f^{*}f(u_{n}-\hat{u})\bigr\Vert +4\mathfrak{Y}_{n}\Vert u_{n}\Vert \Vert u_{n}-v_{n}\Vert , \end{aligned}$$

where $M>0$ satisfying

$$ M\ge \sup_{k} \bigl\{ 2\Vert {-}u_{n}\Vert \bigl\Vert u_{n}-\mathfrak{Z} _{n}f^{*}fu_{n}- \bigl(\hat{u}-\mathfrak{Z}_{n}f^{*}f\hat{u}\bigr)- \mathfrak{Y} _{n}u_{n}\bigr\Vert \bigr\} . $$

From (3.10) and (3.11), we get that

$$\begin{aligned} &\Vert u_{n+1}-\hat{u} \Vert ^{2} \\ &\quad \le (1-\mathfrak{X}_{n})\Vert u_{n}-\hat{u} \Vert ^{2}+ \mathfrak{X}_{n}\Vert v_{n}-\hat{u} \Vert ^{2} \\ &\quad \le \Vert u_{n}-\hat{u}\Vert ^{2}+ \mathfrak{Y}_{n}M-\mathfrak{X} _{n}\Vert u_{n}-v_{n}\Vert ^{2} \\ &\quad\quad {}-\Vert u_{n}-v_{n}\Vert ^{2}+4 \mathfrak{Z}_{n}\Vert u_{n}-v_{n}\Vert \bigl\Vert f^{*}f(u_{n}-\hat{u})\bigr\Vert \\ &\quad \quad {}+4\mathfrak{Y}_{n}\Vert u_{n}\Vert \Vert u_{n}-v_{n}\Vert , \end{aligned}$$

which means that

$$\begin{aligned} \mathfrak{X}_{n}\Vert u_{n}-v_{n} \Vert ^{2} & \le \Vert u_{n+1}-u _{n}\Vert \bigl(\Vert u_{n}-\hat{u} \Vert +\Vert u_{n+1}-\hat{u} \Vert \bigr) \\ &\quad {}+\mathfrak{Y}_{n}M-\mathfrak{X}_{n}\Vert u_{n}-v_{n}\Vert ^{2} \\ &\quad {}-\Vert u_{n}-v_{n}\Vert ^{2}+4 \mathfrak{Z}_{n}\Vert u_{n}-v_{n}\Vert \bigl\Vert f^{*}f(u_{n}-\hat{u})\bigr\Vert \\ &\quad {}+4\mathfrak{Y}_{n}\Vert u_{n}\Vert \Vert u_{n}-v_{n}\Vert . \end{aligned}$$

Since $\lim_{n\rightarrow \infty }\mathfrak{Y}_{n}=0$, $\lim_{n\rightarrow \infty }\Vert u_{n+1}-u_{n} \Vert =0$, and $\lim_{n\rightarrow \infty }\Vert f^{*}fu_{n}-f^{*}f\hat{u}\Vert =0$, we infer that

$$ \lim_{n\rightarrow \infty }\Vert u_{n}-v_{n} \Vert =0. $$

Finally, we show that $u_{n}\rightarrow \hat{u}$. Using the property of the projection $P_{S_{i}}$, we derive that

$$\begin{aligned} &\Vert v_{n}-\hat{u} \Vert ^{2} \\ &\quad =\biggl\Vert P_{S_{i}}\biggl[(1-\mathfrak{Y}_{n})\biggl(u_{n}-\frac{\mathfrak{Z}_{n}}{1-\mathfrak{Y}_{n}}f^{*}fu_{n}\biggr) \biggr] \\ &\quad \quad {}-P_{S_{i}}[\mathfrak{Y}_{n}\hat{u}+(1- \mathfrak{Y}_{n}) \biggl(\hat{u}-\frac{\mathfrak{Z}_{n}}{1-\mathfrak{Y}_{n}}f ^{*}fu_{n} \biggr)\biggr\Vert ^{2} \\ &\quad \le \biggl\langle (1-\alpha ) \biggl(I-\frac{\mathfrak{Z}_{n}}{1-\mathfrak{Y} _{n}}(u_{n}- \hat{u})\biggr)-\mathfrak{Y}_{n}\hat{u},v_{n}-\hat{u} \biggr\rangle \\ &\quad \le (1-\mathfrak{Y}_{n})\Vert u_{n}-\hat{u}\Vert \Vert v_{n}- \hat{u}\Vert +\mathfrak{Y}_{n} \langle \hat{u},\hat{u}-v_{n} \rangle \\ &\quad \le \frac{1-\mathfrak{Y}_{n}}{2}\bigl(\Vert u_{n}-\hat{u}\Vert ^{2}+ \Vert v_{n}-\hat{u}\Vert ^{2}\bigr)+ \mathfrak{Y}_{n} \langle \hat{u}, \hat{u}-v_{n} \rangle, \end{aligned}$$

which is equivalent to

3.12 $$\begin{aligned} &\Vert v_{n}-\hat{u} \Vert ^{2}\le \frac{1-\mathfrak{Y}_{n}}{1+ \mathfrak{Y}_{n}}\Vert u_{n}-\hat{u}\Vert ^{2}+ \frac{2\mathfrak{Y} _{n}}{1-\mathfrak{Y}_{n}} \langle \hat{u},\hat{u}-v_{n} \rangle. \end{aligned}$$

It follows from (3.4) and (3.12) that

3.13 $$\begin{aligned} \Vert u_{n+1}-\hat{u} \Vert & \le (1-\mathfrak{X}_{n})\Vert u_{n}- \hat{u} \Vert + \mathfrak{X}_{n}\Vert v_{n}-\hat{u}\Vert \\ & \le (1-\mathfrak{X}_{n})\Vert u_{n}-\hat{u} \Vert + \mathfrak{X} _{n}\biggl(\frac{1-\mathfrak{Y}_{n}}{1+\mathfrak{Y}_{n}}\Vert u_{n}- \hat{u}\Vert ^{2}+\frac{2\mathfrak{Y}_{n}}{1-\mathfrak{Y}_{n}} \langle \hat{u}, \hat{u}-v_{n} \rangle \biggr) \\ & \le \biggl(1-\frac{2\mathfrak{Y}_{n}\mathfrak{Z}_{n}}{1+\mathfrak{Y}_{n}}\biggr) \Vert u_{n}-\hat{u}\Vert ^{2}+\frac{2\mathfrak{Y}_{n}\mathfrak{Z} _{n}}{1-\mathfrak{Y}_{n}} \langle \hat{u},\hat{u}-v_{n} \rangle. \end{aligned}$$

Since $\frac{\mathfrak{Z}_{n}}{1-\mathfrak{Y}_{n}}\in (0,\frac{2}{ \rho (G*G)})$, we observe that $\mathfrak{Y}_{n}\in (0,\frac{ \mathfrak{Z}_{n}\rho (G*G)}{2})$, then we have

$$ \frac{2\mathfrak{Y}_{n}\mathfrak{Z}_{n}}{1-\mathfrak{Y}_{n}}\in \biggl(0,\frac{2 \mathfrak{Z}_{n}(2-\mathfrak{Z}_{n}\rho (G*G))}{\mathfrak{Z}_{n} \rho (G*G)}\biggr), $$

that is to say,

$$ \frac{2\mathfrak{Y}_{n}\mathfrak{Z}_{n}}{1-\mathfrak{Y}_{n}} \langle \hat{u}, \hat{u}-v_{n} \rangle \le \frac{2\mathfrak{Z}_{n}(2-\mathfrak{Z} _{n}\rho (G*G))}{\mathfrak{Z}_{n}\rho (G*G)} \langle \hat{u}, \hat{u}-v_{n} \rangle. $$

By virtue of $\sum_{n=1}^{\infty }\frac{\mathfrak{X}_{n}}{ \mathfrak{Z}_{n}}<\infty $, $\mathfrak{Z}_{n}\in (0, \frac{2}{\rho (G*G)})$, and $\langle \hat{u},\hat{u}-v_{n} \rangle $ is bounded, we obtain that

$$ \sum_{n=1}^{\infty }\biggl(\frac{2\mathfrak{Z}_{n}(2-\mathfrak{Z} _{n}\rho (G*G))}{\mathfrak{Z}_{n}\rho (G*G)} \langle \hat{u}, \hat{u}-v_{n} \rangle \biggr) \langle \hat{u}, \hat{u}-v_{n} \rangle < \infty, $$

which implies that (see [13])

$$ \sum_{n=1}^{\infty }\frac{2\mathfrak{Y}_{n}\mathfrak{Z}_{n}}{1- \mathfrak{Y}_{n}} \langle \hat{u}, \hat{u}-v_{n} \rangle \le \infty. $$

Moreover,

3.14 $$ \sum_{n=1}^{\infty }\frac{2\mathfrak{Y}_{n}\mathfrak{Z}_{n}}{1- \mathfrak{Y}_{n}} \langle \hat{u},\hat{u}-v_{n} \rangle = \sum_{n=1}^{\infty } \frac{2\mathfrak{Y}_{n}\mathfrak{Z}_{n}}{1+ \mathfrak{Y}_{n}}\frac{1+\mathfrak{Y}_{n}}{1-\mathfrak{Y}_{n}} \langle \hat{u}, \hat{u}-v_{n} \rangle. $$

It follows that all the conditions of Lemma 2.5 are satisfied. Combining (3.13), (3.14), and Lemma 3.2, we can show that $u_{n}\rightarrow \hat{u}$.

Applying the Schrödinger–Itô formula to $\hat{\mathfrak{X}} ^{i+1}\hat{\mathfrak{Y}}^{i+1}$, we have

$$\begin{aligned}& \hat{\mathfrak{X}}_{S}^{i+1}\hat{\mathfrak{Y}}_{S}^{i+1} \\& \quad = \int_{0}^{S}\bigl[\hat{\mathfrak{X}}_{s}^{i+1} \bigl((-f)^{\alpha_{0}}\bigl(s, u _{s}^{i+1} \bigr)-(-f)^{\alpha_{0}}\bigl(s, u_{s}^{i}\bigr)\bigr)+ \hat{\mathfrak{Y}}_{s} ^{i+1}\bigl(b^{\alpha_{0}}\bigl(s, u_{s}^{i+1}\bigr)-b^{\alpha_{0}}\bigl(s, u_{s}^{i}\bigr)\bigr)\bigr]\,ds \\& \quad \leq \int_{0}^{S}\bigl[A^{\alpha 0}\bigl(s,u_{s}^{i+1} \bigr) -A^{\alpha 0}(s,u_{s} {i}),\hat{u}_{s}^{i+1}\bigr]\,d\langle B \rangle _{s}+ \int_{0}^{S}\bigl[\hat{u}_{s}^{i}+A \bigl(s,u_{s} ^{i}\bigr)- A^{\alpha 0} \bigl(s,u_{s}^{i-1}\bigr),\hat{u}_{s}^{i+1} \bigr]\,d\langle B \rangle _{s} \\& \quad \leq \delta \int_{0}^{S}\bigl[\hat{\mathfrak{X}}_{s}^{i+1} \bigl( \hat{\mathfrak{X}}_{s}^{i}-\hat{f}\bigl(s, u_{s}^{i}\bigr)\bigr)+\hat{\mathfrak{Y}} _{s}^{i+1}\bigl(\hat{\mathfrak{Y}}_{s}^{i}+ \hat{b}\bigl(s, u_{s}^{i}\bigr)\bigr)\bigr]\,ds+ \int _{0}^{S}\hat{\mathfrak{X}}_{s}^{i+1}\,d\hat{\mathfrak{K}}_{s}^{i+1} \\& \quad \leq \int_{0}^{S}\bigl[\hat{\mathfrak{X}}_{s}^{i+1} \hat{\mathfrak{Z}} _{s}^{i+1}+\hat{\mathfrak{Y}}_{s}^{i+1} \bigl(\sigma^{\alpha_{0}}\bigl(s, u_{s} ^{i+1}\bigr)- \sigma^{\alpha_{0}}\bigl(s, u_{s}^{i}\bigr)\bigr)+\delta \hat{\mathfrak{Y}} _{s}^{i+1}\bigl(\hat{\mathfrak{Z}}_{s}^{i}+ \sigma \bigl(s, u_{s}^{i}\bigr)\bigr)\bigr]\,d\mathfrak{B}_{s}. \end{aligned}$$

Since $\hat{\mathrm{Y}}_{S}^{i+1}=\Phi^{\alpha_{0}}(\mathfrak{X}_{S} ^{i+1})-\Phi^{\alpha_{0}}(\mathfrak{X}_{S}^{i})+\delta (- \hat{\mathfrak{X}}_{S}^{i}+\Phi (\mathfrak{X}_{S}^{i})-\Phi ( \mathfrak{X}_{S}^{i-1}))$, we have

$$\begin{aligned}& \hat{\mathfrak{X}}_{S}^{i+1}\bigl[\Phi^{\alpha_{0}}\bigl( \mathfrak{X}_{S}^{i+1}\bigr)- \Phi^{\alpha_{0}}\bigl( \mathfrak{X}_{S}^{i}\bigr)\bigr] \\& \quad = \delta \bigl[\hat{\mathfrak{X}}_{S}^{i+1}\hat{ \mathfrak{X}}_{S}^{i}- \hat{\mathfrak{X}}_{S}^{i+1} \bigl(\Phi \bigl(\mathfrak{X}_{S}^{i}\bigr)-\Phi \bigl( \mathfrak{X}_{S}^{i-1}\bigr)\bigr)\bigr] \\& \quad \leq \int^{S}\bigl[\hat{\mathfrak{X}}_{s}^{i+1} \bigl((-f)^{\alpha_{0}}\bigl(s, u_{s}^{i+1} \bigr)-(-f)^{ \alpha_{0}}\bigl(s, u_{s}^{i}\bigr)\bigr)+ \mathrm{Y}_{s}^{i+1}\bigl(b^{\alpha_{0}}\bigl(s, u _{s}^{i+1}\bigr)-b^{\alpha_{0}}\bigl(s, u_{s}^{i}\bigr)\bigr)\bigr]\,ds \\& \quad \leq \delta \int^{S}\bigl[\hat{\mathfrak{X}}_{s}^{i+1} \bigl( \hat{\mathfrak{X}}_{s}^{i}-\hat{f}\bigl(s, u_{s}^{i}\bigr)\bigr)+\hat{\mathfrak{Y}} _{s}^{i+1}\bigl(\hat{\mathfrak{Y}}_{s}^{i}+ \hat{b}\bigl(s, u_{s}^{i}\bigr)\bigr)\bigr]\,ds+ \int _{0}^{S}\hat{\mathfrak{X}}_{s}^{i+1}\,d\hat{\mathfrak{K}}_{s}^{i+1} \\& \quad \leq \int_{0}^{S}\bigl[\hat{\mathfrak{X}}_{s}^{i+1} \hat{\mathfrak{Z}}_{s}^{i+1}+ \hat{\mathfrak{Y}}_{s}^{i+1} \bigl(\sigma^{\alpha_{0}}\bigl(s, u_{s}^{i+1}\bigr)- \sigma^{\alpha_{0}}\bigl(s, u_{s}^{i}\bigr)\bigr)+\delta \hat{\mathfrak{Y}}_{s}^{i+1}\bigl( \hat{\mathfrak{Z}}_{s}^{i}+ \sigma \bigl(s, u_{s}^{i}\bigr)\bigr)\bigr]\,d\mathfrak{B}_{s}. \end{aligned}$$

By (H2), (H3), and Lemma 2.6, we have

$$\begin{aligned}& \bigl[(C-1) \alpha +1\bigr]\bigl\vert \hat{\mathfrak{X}}_{S}^{i+1} \bigr\vert ^{2} \\& \quad \leq \delta (1+C_{1})\bigl\vert \hat{\mathfrak{X}}_{S}^{i+1}\hat{\mathfrak{X}}_{S}^{i}\bigr\vert +(-C\alpha +\alpha -1) \int_{0}^{S} \biggr\vert \hat{\mathfrak{X}}_{s}^{i+1} \bigl\vert ^{2}+ \bigr\vert \mathrm{Y}_{s}^{i+1} \biggr\vert ^{2}\,ds \\& \qquad {} +\delta \int_{0}^{S}\bigl[\bigl\vert \hat{\mathfrak{X}}_{s}^{i+1} \hat{\mathfrak{X}}_{s}^{i}\vert +C_{1} \vert \hat{\mathfrak{X}}_{s}^{i+1}\hat{u}_{s}^{i} \vert + \vert \hat{\mathfrak{Y}}_{s}^{i+1}\hat{ \mathfrak{Y}}_{s}^{i} \bigr\vert +C _{1}\bigl\vert \hat{\mathfrak{Y}}_{s}^{i+1}\hat{u}_{s}^{i} \bigr\vert \bigr]\,ds \\& \qquad {}+(-C \alpha +\alpha -1) \int_{0}^{S}\bigl\vert \hat{u}_{s}^{i+1} \bigr\vert ^{2}\,d\langle B \rangle _{s} \\& \qquad {} +\delta (C_{1}+1) \int_{0}^{S}\bigl\vert \hat{u}_{s}^{i+1} \bigr\vert \bigl\vert \hat{u}_{s}^{i} \bigr\vert \,d\langle B \rangle _{s}+ \int_{0}^{S}\hat{\mathfrak{X}}_{s}^{i+1}\,d\hat{\mathfrak{K}}_{s}^{i+1} \\& \qquad {}+ \int_{0}^{S}\bigl[\hat{\mathfrak{Y}}_{s}^{i+1} \bigl(\sigma^{\alpha_{0}}\bigl(s, u _{s}^{i+1}\bigr)- \sigma^{\alpha_{0}}\bigl(s, u_{s}^{i}\bigr)\bigr)+\delta \hat{\mathfrak{Y}}_{s}^{i+1}\bigl(\hat{\mathfrak{Z}}_{s}^{i}+ \sigma \bigl(s, u _{s}^{i}\bigr)-\sigma \bigl(s, u_{s}^{i-1}\bigr)\bigr)\bigr]\,d\mathfrak{B}_{s} \\& \quad \leq \int^{S}\bigl[\hat{\mathfrak{X}}_{s}^{i+1} \bigl((-f)^{\alpha_{0}}\bigl(s, u_{s}^{i+1} \bigr)-(-f)^{ \alpha_{0}}\bigl(s, u_{s}^{i}\bigr)\bigr)+ \mathrm{Y}_{s}^{i+1}\bigl(b^{\alpha_{0}}\bigl(s, u _{s}^{i+1}\bigr)-b^{\alpha_{0}}\bigl(s, u_{s}^{i}\bigr)\bigr)\bigr]\,ds \\& \quad \leq \delta (1+C_{1})\bigl\vert \hat{\mathfrak{X}}_{S}^{i+1} \hat{\mathfrak{X}}_{S}^{i}\bigr\vert +2\delta (C_{1}+1) \int_{0}^{S} \bigl\vert \hat{u}_{s}^{i+1} \bigr\vert \bigl\vert \hat{u}_{s}^{i} \bigr\vert \,ds \\& \qquad {}+(-C \alpha +\alpha -1)\int_{0}^{S}\bigl\vert \hat{u}_{s}^{i+1}\bigr\vert ^{2}\,d\{B\}_{s}+ \delta (C_{1}+1) \int_{0}^{S}\bigl\vert \hat{u}_{s}^{i+1} \bigr\vert \bigl\vert \hat{u}_{s}^{i} \bigr\vert \,d\{B \}_{s}+ \int_{0}^{S}\hat{\mathfrak{X}}_{s}^{i+1}\,d\hat{\mathfrak{K}} _{s}^{i+1} \\& \qquad {} +\int_{0}^{S}\bigl[\hat{\mathfrak{X}}_{s}^{i+1} \hat{\mathfrak{Z}}_{s} ^{i+1}+\hat{\mathfrak{Y}}_{s}^{i+1} \bigl(\sigma^{\alpha_{0}}\bigl(s, u_{s}^{i+1}\bigr)- \sigma^{\alpha_{0}}\bigl(s, u_{s}^{i}\bigr)\bigr)+\delta \hat{\mathfrak{Y}}_{s}^{i+1}\bigl( \hat{\mathfrak{Z}}_{s}^{i}+ \sigma \bigl(s, u_{s}^{i}\bigr)\bigr)\bigr]\,d\mathfrak{B}_{s}, \end{aligned}$$

where, in the second inequality, we have used the fact that $-C\alpha +\alpha -1\leq 0$.

Taking a Schrödinger expectation on both sides of (3.13) and noticing that the last two terms are Schrödinger martingales (see [14]), we get

3.15 $$\begin{aligned}& \bigl[(C-1) \alpha +1\bigr]\bigl\vert \hat{ \mathfrak{X}}_{S}^{i+1} \bigr\vert ^{2} \\& \quad \leq 2\delta (1+C_{1})\hat{\mathrm{E}}\bigl\vert \hat{ \mathfrak{X}}_{S}^{i+1}\hat{\mathfrak{X}}_{S}^{i} \bigr\vert \\& \quad \quad {}+ 4 \delta (C_{1}+1)\hat{\mathrm{E}}\int_{0}^{S} \bigl\vert \hat{u}_{s}^{i+1}\bigr\vert \bigl\vert \hat{u}_{s}^{i}\bigr\vert \,ds+2\delta (C_{1}+1)\hat{\mathrm{E}} \int_{0}^{S} \bigl\vert \hat{u}_{s}^{i+1}\bigl\vert \bigr\vert \hat{u}_{s}^{i} \bigr\vert \,d\{B \}_{s}. \end{aligned}$$

Moreover, by Lemma 2.5, we have

3.16 $$\begin{aligned}& \bigl[(C-1)\alpha +1\bigr] \biggl[\hat{\mathrm{E}}\bigl\vert \hat{\mathfrak{X}}_{S}^{i+1} \bigr\vert ^{2}+ \underline{ \sigma }^{2}\hat{\mathrm{E}} \int_{0}^{S}\bigl\vert \hat{u}_{s}^{i+1} \bigr\vert ^{2}\,ds\biggr] \\& \quad \leq 2\delta (1+C_{1})\hat{\mathrm{E}}\bigl\vert \hat{ \mathfrak{X}}_{S}^{i+1}\hat{\mathfrak{X}}_{S}^{i} \bigr\vert \\& \quad \quad {}+2 \delta (C_{1}+1) \bigl(2+\overline{\sigma }^{2}\bigr) \hat{\mathrm{E}} \int_{0}^{S}\bigl\vert \hat{u}_{s}^{i+1} \bigr\vert \bigl\vert \hat{u}_{s}^{i} \bigr\vert \,ds. \end{aligned}$$

Let

$$ C_{2} := \min \bigl((C-1)\alpha +1, \underline{\sigma }^{2} \bigl[(C-1)\alpha +1\bigr]\bigr) $$

and

$$ C_{3}:=2(C_{1}+1) \bigl(2+\overline{\sigma }^{2} \bigr). $$

We have

$$\begin{aligned} \hat{\mathrm{E}}\bigl\vert \hat{\mathfrak{X}}_{S}^{i+1} \bigr\vert ^{2}+\hat{\mathrm{E}} \int_{0}^{S}\bigl\vert \hat{u}_{s}^{i+1} \bigr\vert ^{2}\,ds\leq \frac{C_{3}\delta }{C_{2}}\biggl[ \hat{\mathrm{E}}\bigl\vert \hat{\mathfrak{X}}_{S}^{i+1}\bigr\vert \bigl\vert \hat{\mathfrak{X}}_{S}^{i}\bigr\vert +\hat{\mathrm{E}} \int_{0}^{S} \bigl\vert \hat{u}_{s}^{i+1} \bigr\vert \bigl\vert \hat{u}_{s}^{i}\bigr\vert \,ds\biggr]. \end{aligned}$$

By Young’s inequality, we get

$$\begin{aligned} \hat{\mathrm{E}}\bigl\vert \hat{\mathfrak{X}}_{S}^{i+1} \bigr\vert ^{2}+\hat{\mathrm{E}} \int_{0}^{S}\bigl\vert \hat{u}_{s}^{i+1} \bigr\vert ^{2}\,ds\leq \biggl(\frac{C_{3}}{C_{2}} \biggr)^{2} \delta^{2}\biggl[\hat{\mathrm{E}}\bigl\vert \hat{ \mathfrak{X}}_{S}^{i} \bigr\vert ^{2}+ \hat{ \mathrm{E}} \int_{0}^{S}\bigl\vert \hat{u}_{s}^{i} \bigr\vert ^{2}\,ds\biggr]. \end{aligned}$$

Notice that

$$\begin{aligned} \hat{\mathfrak{X}}_{S}^{i} =& \int_{0}^{S}\bigl[b^{\alpha_{0}}\bigl(s, u_{s} ^{i}\bigr)-b^{\alpha_{0}}\bigl(s, u_{s}^{i-1}\bigr)+\delta \bigl(\hat{\mathfrak{Y}}_{s} ^{i}+b\bigl(s, u_{s}^{i-1}\bigr)-b\bigl(s, u_{s}^{i-2}\bigr)\bigr)\bigr]\,ds \\ &+ \int_{0}^{S}\bigl[h^{\alpha_{0}}\bigl(s, u_{s}^{i}\bigr)-h^{\alpha_{0}}\bigl(s, u _{s}^{i-1}\bigr)+\delta \bigl(\hat{\mathfrak{Y}}_{s}^{i}+h \bigl(s, u_{s}^{i-1}\bigr)-h\bigl(s, u_{s}^{i-2} \bigr)\bigr)\bigr]\,d\langle B \rangle _{s} \\ &+ \int_{0}^{t}\bigl[\sigma^{\alpha_{0}}\bigl(s, u_{s}^{i}\bigr)-\sigma^{\alpha_{0}}\bigl(s, u_{s}^{i-1}\bigr)+\delta \bigl(\hat{\mathfrak{Y}}_{s}^{i}+ \sigma \bigl(s, u_{s} ^{i-1}\bigr)-\sigma \bigl(s, u_{s}^{i-2}\bigr)\bigr)\bigr]\,d\mathfrak{B}_{s}. \end{aligned}$$

By (H1)–(H3), Lemmas 2.3 and 2.5, and a standard method of estimation, we can derive easily that there exists a positive constant $C_{4}$ which depends only on *C*, $\overline{\sigma }^{2}$ such that

$$ \hat{\mathrm{E}}\bigl\vert \hat{\mathfrak{X}}_{S}^{i} \bigr\vert ^{2}\leq C_{4}\biggl[ \hat{\mathrm{E}} \int_{0}^{S}\bigl\vert \hat{u}_{s}^{i} \bigr\vert ^{2}\,ds+\hat{\mathrm{E}} \int_{0}^{S}\bigl\vert \hat{u}_{s}^{i-1} \bigr\vert ^{2}\,ds\biggr], \quad \forall i\geq 1. $$

So there exists a positive constant $C_{5}$ which depends on *C*, $C_{1}$, $\underline{\sigma}^{2}$, and $\overline{\sigma }^{2}$ such that

$$\begin{aligned} \hat{\mathrm{E}} \int_{0}^{S}\bigl\vert \hat{u}_{s}^{i+1} \bigr\vert ^{2}\,ds\leq C_{5}\delta ^{2}\biggl[ \hat{\mathrm{E}} \int_{0}^{S}\bigl\vert \hat{u}_{s}^{i} \bigr\vert ^{2}\,ds + \hat{\mathrm{E}} \int_{0}^{S}\bigl\vert \hat{u}_{s}^{i-1} \bigr\vert ^{2}\,ds\biggr]. \end{aligned}$$

It follows that there exists $\delta_{0}\in (0,1)$ which depends only on *C*, $C_{1}$, $\underline{\sigma}^{2}$, and $\overline{\sigma } ^{2}$ such that, when $0<\delta \leq \delta_{0}$,

$$\begin{aligned} \hat{\mathrm{E}} \int_{0}^{S}\bigl\vert \hat{u}_{s}^{i+1} \bigr\vert ^{2}\,ds\leq \frac{1}{4} \hat{\mathrm{E}} \int_{0}^{S}\bigl\vert \hat{u}_{s}^{i} \bigr\vert ^{2}\,ds+\frac{1}{8} \hat{\mathrm{E}} \int_{0}^{S}\bigl\vert \hat{u}_{s}^{i-1}\bigr\vert ^{2}\,ds. \end{aligned}$$

By Lemma 2.5, it turns out that $u^{i}$ is a Cauchy sequence in $M_{G}^{2}(0, S)$. We denote its limit by $u^{\alpha }=( \mathfrak{X}^{\alpha },\mathfrak{Y}^{\alpha },\mathfrak{Z}^{\alpha })$.

Now, we deal with the sequence $\mathfrak{K}^{i}$, $i=0,1,2, \ldots $ ,

$$\begin{aligned} \hat{\mathfrak{K}}_{s}^{i+1} =& \Phi^{\alpha_{0}}\bigl( \mathfrak{X}_{s} ^{i+1}\bigr)-\Phi^{\alpha_{0}}\bigl( \mathfrak{X}_{s}^{i}\bigr)+\delta \bigl(- \hat{ \mathfrak{X}}_{s}^{i} +\Phi \bigl(\mathfrak{X}_{s}^{i} \bigr)-\Phi \bigl( \mathfrak{X}_{s}^{i-1}\bigr)\bigr)+\hat{ \mathfrak{Y}}_{s}^{i+1} \\ &+ \int_{0}^{t}\bigl[(-f)^{\alpha_{0}}\bigl(s, u_{s}^{i+1}\bigr)-(-f)^{\alpha_{0}}\bigl(s, u_{s}^{i}\bigr)+\delta \bigl(\hat{\mathfrak{X}}_{s}^{i}- \hat{f}\bigl(s, u_{s}^{i}\bigr)\bigr)\bigr]\,ds \\ &+ \int_{0}^{s}\bigl[(-g)^{\alpha_{0}}\bigl(s, u_{s}^{i+1}\bigr)-(-g)^{\alpha_{0}}\bigl(s, u_{s}^{i}\bigr)+\delta \bigl(\hat{\mathfrak{X}}_{s}^{i}- \hat{g}\bigl(s, u_{s}^{i}\bigr)\bigr)\bigr]\,d\mathfrak{X}^{\alpha }_{s} \\ &+ \int_{0}^{s}\hat{\mathfrak{Z}}_{s}^{i+1}\,d\mathfrak{B}_{s}. \end{aligned}$$

By (H2) and $u^{i}$ is a Cauchy sequence in $M_{G}^{2}(0, S)$, it is easy to get that $\mathfrak{K}^{i}$ is a Cauchy sequence in $L_{G}^{2}(\Gamma_{s})$. We denote its limit by $\mathfrak{K}^{\alpha }$, which is a decreasing process with $\mathfrak{K}_{0}^{\alpha }=0$ and $\mathfrak{K}_{S}^{\alpha }\in L_{G}^{2}(\Gamma_{S})$.

Taking limit in (3.10), we get that, when $0<\delta \leq \delta _{0}, (u^{\alpha }, \mathfrak{K}^{\alpha })=(\mathfrak{X}^{\alpha }, \mathfrak{Y}^{\alpha }, \mathfrak{Z}^{\alpha }, \mathfrak{K}^{ \alpha })$ satisfies (1.1) for $\alpha =\alpha_{0}+\delta $. □

## Conclusions

By using new Schrödinger type inequalities appearing in Jiang and Usó [1], we studied the existence of weak solutions of stochastic delay differential systems with Schrödinger–Brownian motions. By using the continuation theorem of coincidence degree theory and the method of Schrödingerean Lyapunov functional, some sufficient conditions were obtained.
